# Basic Study of Drug-Drug Interaction between Memantine and the Traditional Japanese Kampo Medicine Yokukansan

**DOI:** 10.3390/molecules24010115

**Published:** 2018-12-29

**Authors:** Takashi Matsumoto, Kyoji Sekiguchi, Zenji Kawakami, Junko Watanabe, Kazushige Mizoguchi, Yasushi Ikarashi, Masahiro Yamamoto

**Affiliations:** Tsumura Kampo Research Laboratories, Kampo Research & Development Division, Tsumura & Co., 300-1192 Ibaraki, Japan; sekiguchi_kyouji@mail.tsumura.co.jp (K.S.); kawakami_zenji@mail.tsumura.co.jp (Z.K.); watanabe_junko@mail.tsumura.co.jp (J.W.); mizoguchi_kazushige@mail.tsumura.co.jp (K.M.); ikarashi_yasushi@mail.tsumura.co.jp (Y.I.); yamamoto_masahiro@mail.tsumura.co.jp (M.Y.)

**Keywords:** drug-drug interaction, memantine, yokukansan, NMDA receptor, pharmacokinetics, pharmacology

## Abstract

Several basic pharmacokinetic and pharmacological studies were conducted as part of a group of studies to clarify the drug-drug interaction (DDI) between memantine (MEM), a drug used to treat Alzheimer’s disease, and yokukansan (YKS), a traditional Japanese Kampo medicine used to treat behavioral and psychological symptoms of dementia. The pharmacokinetic studies showed that there were no statistically significant differences in MEM concentrations in the plasma, brain, and urine between mice treated with MEM alone and with MEM plus YKS. Regarding candidate active ingredients of YKS, there were also no statistically significant differences in concentrations of geissoschizine methyl ether in the plasma and brain, urine, glycyrrhetinic acid in the plasma, and isoliquiritigenin in the urine, in mice treated with YKS alone or with MEM plus YKS. The pharmacological studies showed that isoliquiritigenin, which has an *N*-methyl-d-aspartic acid (NMDA) receptor antagonistic effect, did not affect the inhibitory effect of MEM on NMDA-induced intracellular Ca^2+^ influx in primary cultured rat cortical neurons. Moreover, YKS did not affect either the ameliorative effects of MEM on NMDA-induced learning and memory impairment, or the MEM-induced decrease in locomotor activities in mice. These results suggest that there is probably no pharmacokinetic or pharmacological interaction between MEM and YKS in mice, but more detailed studies are needed in the future. Our findings provide important information for future studies, to clarify the DDI more regarding the efficacy and safety of combined use of these drugs in a clinical situation.

## 1. Introduction

Memantine (MEM) hydrochloride, a therapeutic agent used to treat Alzheimer’s disease, was developed by Merz Pharmaceuticals GmbH in Germany [1], and was approved in the European Medicines Agency in 2002, the U.S. Food and Drug Administration in 2003, and the Japanese Ministry of Health, Labour and Welfare (MHLW) in 2011 for the treatment of patients with moderate-to-severe dementia from Alzheimer’s disease [1]. Various in vitro tests determined the mechanism of action to be a voltage-dependent, uncompetitive *N*-methyl-d-aspartic acid (NMDA) receptor antagonist, in which MEM showed low-to-moderate affinity and a fast binding–dissociation rate with the receptor [1]. In addition, MEM has ameliorative actions against excessive glutamate-induced neurocytotoxicity without affecting the physiological glutamate neuronal activity [1,2,3]. In humans, MEM has been reported to modify the progressive symptomatic decline in cognition, function, and behavior in patients with moderate-to-severe Alzheimer’s disease in 12- to 28-week trials [4,5]. Animal studies using rats have demonstrated that MEM protects cognitive dysfunction induced by the sequential injection of β-amyloid and ibotenate into the bilateral hippocampus [6], NMDA-induced impairment of passive avoidance learning and long-term potentiation formation [7], and neurodegeneration induced by β-amyloid [8]. On the other hand, high doses of MEM inhibit motor activity [9].

Yokukansan (YKS) is composed of seven crude drugs (see “Materials and Methods” section) and is the traditional Japanese Kampo medicine approved by the Japanese MHLW. It has indications for the relief of the following symptoms of patients with delicate constitutions and nervousness: neurosis, insomnia, and night crying and peevishness in children. Recently, it has been reported that YKS improves the behavioral and psychological symptoms of dementia (BPSD), such as aggressiveness, irritability, and hallucinations observed in patients with various types of dementia [10,11,12]. YKS has pharmacological mechanisms in several neurotransmitter systems, including serotonergic, glutamatergic, dopaminergic, GABAergic, and cholinergic systems [13,14,15]. In particular, YKS was demonstrated to decrease excessive glutamate concentrations in the synaptic clefts, which may be mediated by reducing glutamate release from presynaptic sites and enhancing glutamate uptake into astrocytes via glutamate transporter activation [16,17]. Recently, YKS was reported to normalize stress-induced decreases in glutamate transporter expression in the mouse hippocampus [18]. YKS also has protective effects against glutamate-induced cell death in cultured neurons [19]. In addition, several active ingredients involving the glutamatergic system have been identified. For example, Glycyrrhiza-derived isoliquiritigenin (ILQG) was demonstrated to have NMDA receptor antagonistic action and mediate the suppression of intracellular Ca^2+^ influx similar to MEM [20]. Glycyrrhetinic acid (GA), a main metabolite of glycyrrhizic acid derived from Glycyrrhiza, was reported to normalize the reduced expression of glutamate transporters in cultured astrocytes subjected to thiamine deficiency [16]. Furthermore, geissoschizine methyl ether (GM), found in Uncaria hooks, has a partial agonistic action on serotonin 1A receptors, and is a candidate for the psychopharmacological effect of YKS [21], as well as GA.

A combination of MEM and YKS is sometimes used clinically to comprehensively treat dementia, as these drugs are applied individually to treat cognitive deficits and BPSD, respectively. In addition, recently MEM has also been used in the treatment of neuropsychiatric disorders (bipolar mood disorders, major depression, schizophrenia and psychotic disorders, anxiety disorders, attention-deficit hyperactivity disorder, etc.) other than dementia [22]. YKS has also been used for the symptomatic treatment of other psychiatric disorders (schizophrenia, personality disorders, tardive dyskinesia, delirium and anxiety disorders, pervasive developmental disorders including Asperger’s disorder, etc.) other than dementia [13,14]. These findings suggest that even in neuropsychiatric disorders other than dementia, there is a possibility that two drugs may be used together in a clinical situation. In such cases, information regarding efficacy and safety is required by medical practitioners. As previously mentioned, both drugs have a similar course of action to reduce glutamatergic hyperactivation in the brain; therefore, a risk of drug-drug interactions (DDIs) affecting the efficacy and safety is assumed when used in combination. DDIs that may result in an altered therapeutic response include pharmacokinetic drug interactions and pharmacological drug interactions [23]. The former influences the processes of absorption, distribution, metabolism, and excretion (ADME), resulting in a change in drug concentration at the site of action. The latter strengthens or attenuates the pharmacological action of the drug when one drug affects the action of another, which is a different mechanism from ADME.

Previous pharmacokinetic studies into MEM [24] and YKS [25,26] have shown that MEM and some of the active ingredients in YKS, such as GM and GA, are absorbed into the blood after oral administration, and distributed to the brain, which is a target organ for both drugs. MEM is hardly metabolized, and its first-pass effect is low in humans; therefore, about 80% of circulating MEM-related material is present as the parent compound [27]. In vitro studies using human liver cells or liver microsomes have shown that MEM does not induce or inhibit drug-metabolizing enzymes [1]. MEM is almost entirely excreted in the urine via organic cation transporter 2 [28]. On the other hand, not all metabolites of ingredients in YKS are known, and in vitro studies using rat and human liver microsomes have reported that GM is metabolized into several metabolites in both species [29,30]. Furthermore, cytochrome P450 (CYP) 3A4 was shown mainly to contribute to GM metabolism in human liver microsomes [30]. In a clinical study, seven-day continuous administration of YKS did not affect the activity of CYP1A2, CYP2D6, CYP3A, xanthine oxidase, or *N*-acetyltransferase 2 [31]. GM was also reported to be excreted in the urine [32]; however, it is not known whether the ingredients of YKS, including GM, are substrates of their transporters, and whether they induce or inhibit the transporters.

Thus, while the individual pharmacokinetic and pharmacological properties of each drug have been studied, the pharmacokinetic and pharmacological drug interactions involved when both drugs are used together remain unknown. Accordingly, we considered that the DDI between MEM and YKS should be investigated for the appropriate use for the treatment of dementia. In the present study, we performed basic pharmacokinetics and pharmacology studies as part of studies to clarify the DDI between the two drugs. In the pharmacokinetic study, the concentrations of MEM and YKS-derived bioactive ingredients (GM, GA, and ILQG) in the plasma, brain, and urine were measured in mice after oral administration of MEM, YKS, or a combination of the two drugs. In the pharmacological study, DDIs were evaluated by measuring NMDA-induced intracellular Ca^2+^ influx in rat primary cultured neurons, as well as in NMDA-induced learning and memory impairment and locomotor activity inhibition in mice.

## 2. Results and Discussion

### 2.1. Pharmacokinetic Interaction

MEM is a single chemical compound, whereas YKS is composed of seven crude drugs, and at least 25 ingredients that have been identified by three-dimensional liquid chromatographic analysis with ultraviolet detection [14]. Among these ingredients, GM [21] and GA [16] are predicted to be active ingredients responsible for the ameliorative effect of YKS on BPSD-like phenotypes, and ILQG [20] has an antagonistic action against NMDA receptors similar to that of MEM. Therefore, we performed pharmacokinetic studies to evaluate the DDI between MEM and the three YKS-derived ingredients.

#### 2.1.1. Plasma and Brain Concentrations of Memantine and Yokukansan-Derived Ingredients

**Figure 1:**
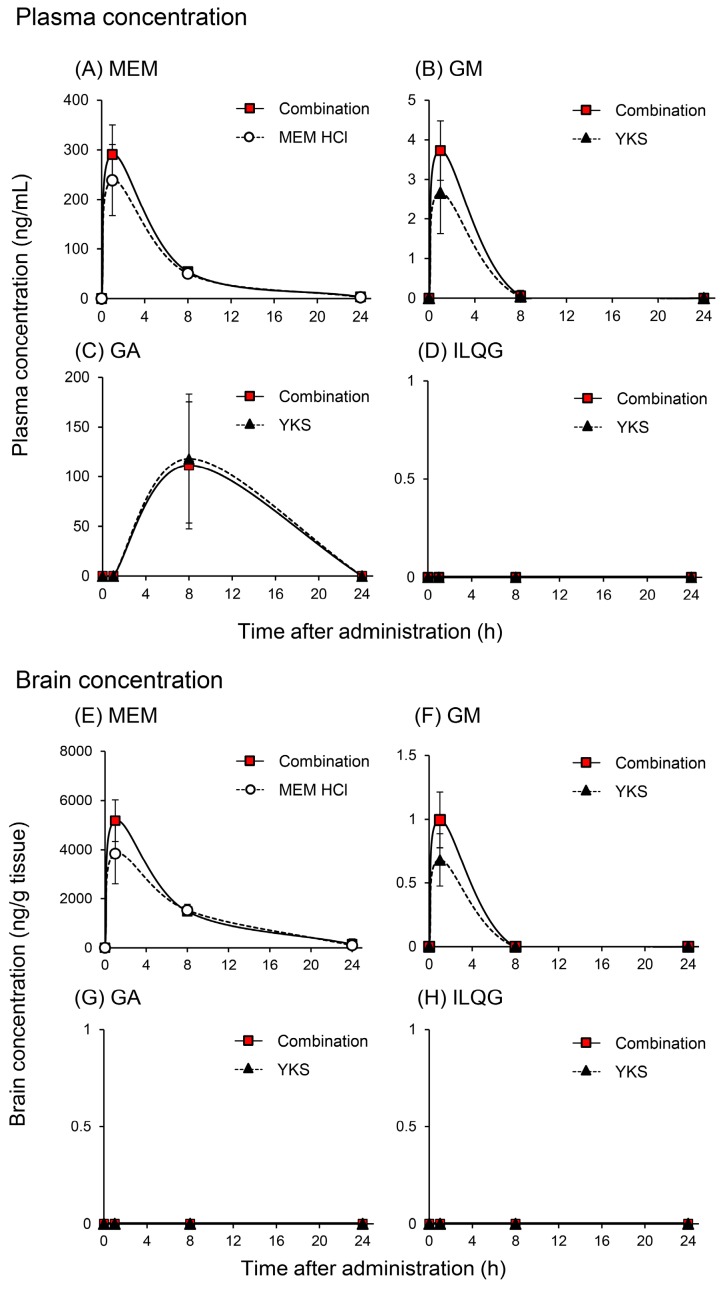
Concentration–time profiles of memantine (MEM) and the three yokukansan (YKS)-derived ingredients in the plasma (**A**–**D**) and brain (**E**–**H**) of mice after oral administration of MEM, YKS, or their combination. MEM hydrochloride (5 mg/kg), YKS (1 g/kg), or their combination was orally administered to fasted mice. Plasma and brain samples were obtained at 0 (pre-administration), 1, 8, or 24 h after administration. Data represent mean ± standard deviation (*n* = 4,5). No statistical significance was observed between the MEM group, YKS group, or the combination group (two-way ANOVA).

Figure 1 shows the changes in the concentrations of MEM and the three YKS-derived ingredients in the plasma and brain of mice orally administered with MEM, YKS, or their combination. MEM concentrations in the plasma (Figure 1A) and brain (Figure 1E) of mice administered with 5 mg/kg MEM hydrochloride (equivalent to the clinical dose in human) reached a maximum at 1 h after administration, and almost disappeared after 24 h. These results partially agree with a previous study demonstrating that the elimination half-life of MEM in the plasma is very fast in mice [24]. Compared with the MEM-alone group, the peak concentration of MEM in the plasma (Figure 1A) and brain (Figure 1E) of the 1 g/kg YKS (this dosage is an equivalent dose showing neuropharmacological actions in rodents) combined group tended to be higher (approximately 20% and 35% in the plasma and brain). However, the time-dependent changes in MEM concentrations between both groups were not statistically significant (two-way ANOVA; group factor: *F*_1, 26_ = 1.76, *p* = 0.196 in the plasma; and *F*_1, 26_ = 3.11, *p* = 0.090 in the brain). In addition to this result, MEM has been demonstrated to be hardly metabolized by human CYP in studies using recombinant CYP isoforms; however, a part of it was metabolized to the gludantan form (furanose type glucuronic acid conjugate), 6-hydroxy form, and 4-hydroxy form [1]. YKS did not affect the induction and inhibition of CYPs and non-CYP enzymes [31,33,34,35]. Combined with these findings, the present results infer that plasma and brain concentrations of MEM may not be affected by co-treatment with YKS, although more detailed studies are necessary in the future.

GM concentrations in the plasma (Figure 1B) and brain (Figure 1F) of mice orally administered with 1 g/kg YKS reached a maximum at 1 h after administration, and almost disappeared around 8–24 h (quantification limit: 0.200 ng/mL in the plasma or 0.500–1.00 ng/g tissue weight in the brain). These results are in agreement with those reported previously [25,36]. Compared with the YKS-alone group, the peak concentrations of GM in the plasma (Figure 1B) and brain (Figure 1F) of the MEM + YKS-combined group tended to be increased (approximately 40% and 40% in the plasma and brain), although the time-dependent changes in GM concentrations between both groups were not statistically significant (two-way ANOVA, group factor: *F*_1, 25_ = 2.98, *p* = 0.096 in the plasma; and *F*_1, 25_ = 4.09, *p* = 0.054 in the brain). In addition to this result, GM has been reported to be metabolized into several metabolites in rats and humans [29,30], and its metabolism is performed mainly by CYP3A4 in humans [30]. On the other hand, an in vitro study has been showed that MEM does not induce or inhibit the activity of CYPs, epoxide hydrolase, flavin-containing monooxygenase, and UDP-glucuronosyltransferase [1]. Combined with these findings, the present results infer that the GM profile in the plasma and brain after administration of YKS may not be affected by co-treatment with MEM, although more detailed studies will be necessary in the future.

GA was only detected in plasma (Figure 1C), and was not found in the brain (Figure 1G) after oral administration of 1 g/kg YKS. Plasma GA concentrations reached a maximum at 8 h after administration, and gradually decreased to nearly below the quantification limit (2.00 ng/mL) until 24 h. These results partially agree with those previously found in rats [26] and humans [37]. GA was not detected in the mouse brain in the present study; however, Tabuchi et al. [26] detected GA in the brains of rats treated with 0.5–2 g/kg YKS. Although we cannot fully explain this discrepancy, it may be due to species differences and the analytical sensitivity for GA. GA is a main metabolite of glycyrrhizin (or glycyrrhizic acid, a glycoside of GA) derived from Glycyrrhiza. Oral glycyrrhizin is poorly absorbed from the intestine because it is a glycoside and has high polarity, most of which is absorbed as GA after presystemic hydrolysis by bacteria [38]. GA absorbed into the blood is taken up into the liver and metabolized into glucuronide and sulfate conjugates, and then excreted into the intestine via the bile [39]. It conjugates back to GA by the intestinal bacteria and is reabsorbed into the blood from the large intestine [14,40]. Thus, the prolonged length of time taken to reach the maximum concentration may be explained by the involvement of intestinal bacterial metabolism and enterohepatic circulation. Figure 1C shows that the concentration–time profile of GA after YKS administration was not significantly affected (two-way ANOVA, group factor: *F*_1, 25_ = 0.01, *p* = 0.905) by co-treatment with 5 mg/kg MEM hydrochloride. This result suggests that MEM does not affect the intestinal bacterial metabolism and enterohepatic circulation of GA.

ILQG was demonstrated to be detected in both the plasma and brain of rats administered with 1 g/kg rikkunshito, another Kampo medicine containing Glycyrrhiza [41]. However, in the present study, this ingredient could not be detected in the plasma and brain; therefore, we could not evaluate the DDI between MEM and ILQG.

Taken together, the results showing that the changes in concentrations of MEM and the major ingredients in YKS in the plasma and brain were not statistically significant between MEM or YKS alone and their combination groups, suggesting that use of the two drugs in combination probably does not lead to DDI.

#### 2.1.2. Urine Concentrations of Memantine and Yokukansan-Derived Ingredients

**Figure 2:**
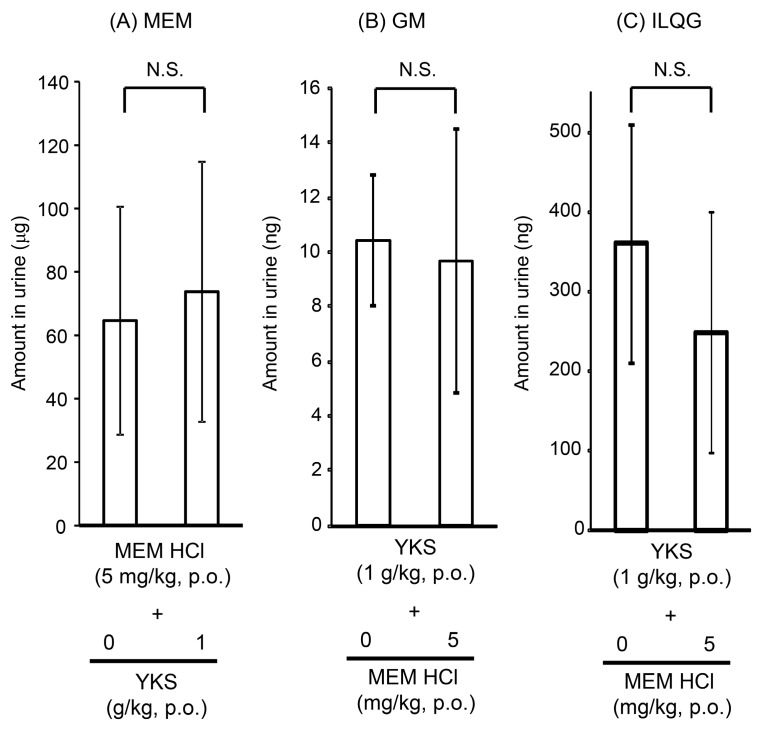
Excretion of MEM (**A**), GM (**B**), and ILQG (**C**) in urine from mice after oral administration of MEM, YKS, or their combination. Urine samples were collected for 24 h after administration of MEM hydrochloride (5 mg/kg), YKS (1 g/kg), or their combination in fasted mice. Data represent mean ± standard deviation (*n* = 4,5). No statistical significances were observed between MEM or YKS group and the combination group (Student’s *t*-test).

Excretion of MEM and two of the YKS-derived ingredients (GM and ILQG) in urine collected during 24 h from mice orally administered with MEM hydrochloride (5 mg/kg), YKS (1 g/kg), or their combination is shown in Figure 2.

Urinary excretion of MEM after oral administration was 65 μg (Figure 2A), which corresponded to approximately 50% of the administered dose. MEM was suggested to be actively transported into the urine involving organic cation transporter 2 and multidrug and toxin extrusion protein 1 [28,42]. Therefore, an inhibitory effect on the drug transporters may result in altered plasma MEM levels. As described above, the plasma MEM profile was not affected by co-treatment with YKS (Figure 1A), and the urinary excretion of MEM was not also significantly affected by co-treatment with YKS (*p* = 0.719). These results suggest that YKS does not affect urinary excretion of MEM.

GM (Figure 2B) and ILQG (Figure 2C) were detected in the urine of YKS-treated mice. Urinary excretion of GM (*p* = 0.796) or ILQG (*p* = 0.304) did not show significant changes when MEM was administered in combination. In agreement with a previous report [43], GA was not detected in the urine, suggesting that plasma GA is mainly excreted in bile, but not in urine. Most MEM absorbed into the blood was demonstrated to be excreted from the kidney into urine as an unchanged form, without being metabolized by CYPs [24]. On the other hand, the metabolism and excretion of GM and ILQG have not been completely elucidated, but our results demonstrating the excretion of both ingredients into urine are similar to those reported previously [32,44]. To excrete orally administered ingredients into the urine, the ingredients must first be absorbed into the blood. Detection of ILQG in the urine (Figure 2C) after oral administration of YKS suggests that this ingredient was absorbed into the blood, although it was not detected in the plasma (Figure 1D).

Excretion of GM or ILQG into the urine after oral administration of YKS did not show any significant change with co-administration of MEM. This result suggests that the excretion process into the urine from the blood of both ingredients is not affected by combination treatment with MEM.

Taken together, the results of the urinary analysis suggest that, similar to the plasma and brain analyses, there is probably no DDI between MEM and YKS when used in combination.

### 2.2. Pharmacological Interaction

#### 2.2.1. *N*-Methyl-d-Aspartic Acid-Induced Intracellular Ca^2+^ Influx

**Figure 3:**
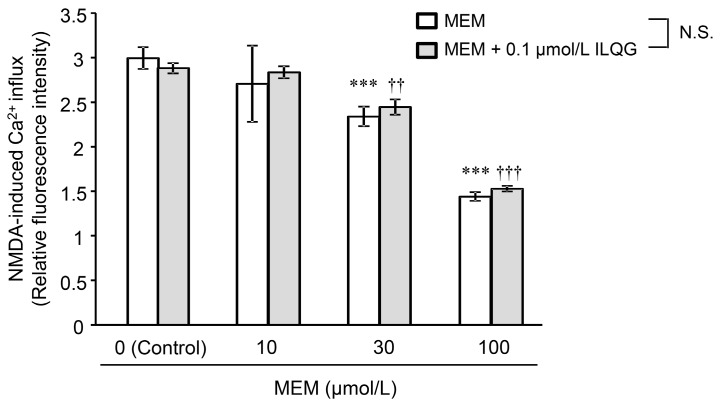
Inhibitory effects of MEM, isoliquiritigenin (ILQG), and their combination on NMDA-induced Ca^2+^ influx in primary cultured neurons. A 3-min, NMDA-induced Ca^2^^+^ influx reaction was started by adding recording buffer containing NMDA (final concentration of 300 μmol/L) or NNDA + test substance (final concentration of 10–100 μmol/L MEM, 0.1 μmol/L ILQG, or a mixture) to cultured cells treated with loading buffer, including Fluo 4-AM (5 μmol/L) at 37 °C for 60 min. Fluorescence intensities of Fluo 4 were measured before (pre-value) and 3 min after the addition of the recording buffer using a plate reader. The Ca^2^^+^ influx data for 3 min are expressed as relative fluorescence intensity against the pre-value before the addition of the recording buffer including NMDA, and are represented as mean ± standard deviation (*n* = 6). *** *p* < 0.001 versus control (vehicle) in MEM group, and ^††^ *p* < 0.01 and ^†††^ *p* < 0.001 versus control (0.1 μmol/L ILQG) in the MEM + ILQG group (Tukey’s test following two-way ANOVA). N.S.: no statistical significance was observed between MEM and MEM + ILQG groups.

The combined effects of MEM and ILQG on NMDA-induced intracellular Ca^2+^ influx in primary cultured neurons are shown in Figure 3 (two-way ANOVA: group factor, *F*_1, 40_ = 1.15, *p* = 0.289; dose factor: *F*_3, 40_ = 177.46, *p* < 0.001; group × dose interaction: *F*_3, 40_ = 1.29, *p* = 0.291). MEM (10–100 μmol/L) inhibited the intracellular Ca^2+^ influx in a concentration-dependent manner. ILQG (0.1 μmol/L) did not inhibit Ca^2+^ influx (control in MEM + ILQG group), and did not affect the inhibitory effect of MEM in combination with each concentration of MEM.

We reported that ILQG binds to NMDA receptors and inhibits NMDA-induced Ca^2+^ influx at high concentrations of 100–300 μmol/L [20]. However, as shown in Figure 1, ILQG was not detected in the plasma and brain of mice orally administered YKS, suggesting that the concentration of ILQG into the brain might be very low (below the quantification limit of 9.8 pmol/g tissue). Although the ILQG concentration (0.1 μmol/L) used in the present study was approximately 10-fold higher than presumptive concentrations in the plasma and brain, it did not inhibit Ca^2+^ influx, and also did not enhance the inhibitory effect of MEM. Thus, this result suggests that there is likely no DDI between MEM and ILQG on inhibition of NMDA-induced Ca^2+^ influx when used in combination.

#### 2.2.2. Learning and Memory Functions

**Figure 4:**
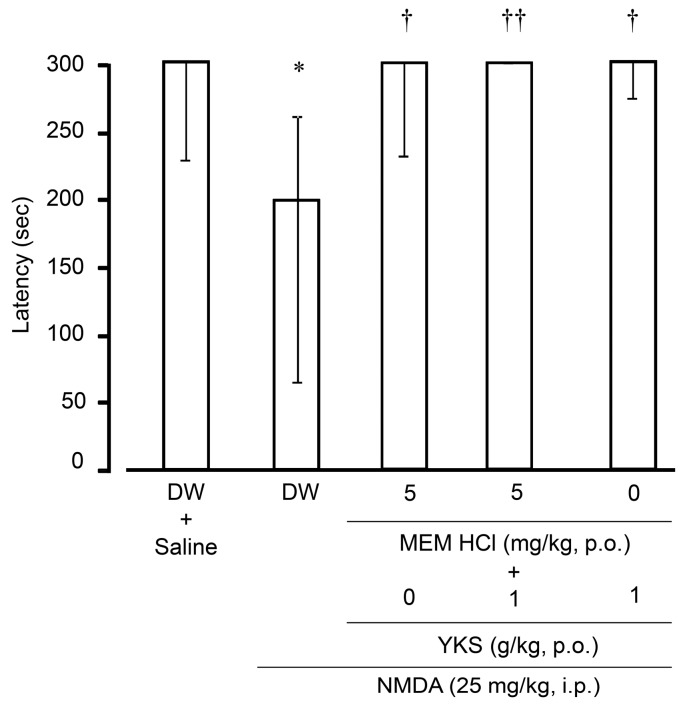
Effects of MEM, YKS, and their combination on NMDA-induced learning and memory disturbances in a step-through passive avoidance task. MEM hydrochloride (5 mg/kg), YKS (1 g/kg), their combination, or distilled water (DW; 10 ml/kg used as a control) was orally administered to mice once a day for 14 days. An acquisition trial was then performed 1 h after the final administration. NMDA (25 mg/kg) or saline (10 mL/kg as control) was injected intraperitoneally 30 min before the trial. After 24 h, a retention trial was performed. The cut-off time of the latency was set at 300 s. Data represent median ± interquartile range (*n* = 9). * *p* < 0.05 versus vehicle control: Wilcoxon rank-sum test, ^†^ *p* < 0.05 and ^††^ *p* < 0.01 versus NMDA (Dunnett’s test following one-way ANOVA).

The effects of MEM, YKS, and their combination on NMDA-induced learning and memory disturbances in mice were examined by a step-through passive avoidance test (Figure 4, one-way ANOVA: *F*_3, 32_ = 8.45, *p* < 0.001). In the control mice, the median latency time in the retention trial performed 24 h after the acquisition trial was 300 s, suggesting that the avoidance memory obtained in the acquisition trial was retained for 24 h. However, the latency time in the retention trial was significantly shortened by administering NMDA 30 min before the acquisition trial, suggesting that learning and memory functions were impaired by NMDA. The shortened latency time returned to 300 s by pretreatment with YKS or MEM for 14 days. These results suggest that YKS and MEM prevent NMDA-induced learning and memory disturbances.

A prolonged latency time is well known to be affected by drug-dependent physical effects, such as catalepsy and suppression of motor activity [45]. We confirmed that the doses of YKS (1 g/kg) and MEM hydrochloride (5 mg/kg) used in the present study did not affect locomotor activity. Therefore, the ameliorative effects of YKS and MEM on the shorter latency in NMDA-treated mice are not thought to be due to physical disturbances—that is, the changes are selective to memory function.

The learning and memory improving effects of MEM are known to be conversely attenuated when the NMDA receptor inhibitory action is markedly enhanced (e.g., at high doses) [7,46]. Considering that YKS can reduce glutamatergic signaling, YKS was considered to affect the learning and memory improving effects of MEM, even though the action mechanisms of both drugs may differ. However, in the present study, the improving effect of MEM was not modulated by co-treatment with YKS, suggesting that YKS does not enhance the inhibitory effects of MEM on the glutamatergic system, and does not interfere with the improvement effect of MEM. YKS was reported to improve learning and memory disturbances as well as BPSD-like behaviors in various animal models of dementia [13]. Its mechanism is thought not to be a direct NMDA receptor inhibitory action like MEM, but an additive or synergic action, including neuroprotective and modulatory effects on several neurotransmissions by various ingredients [14].

#### 2.2.3. Locomotor Activity

**Figure 5:**
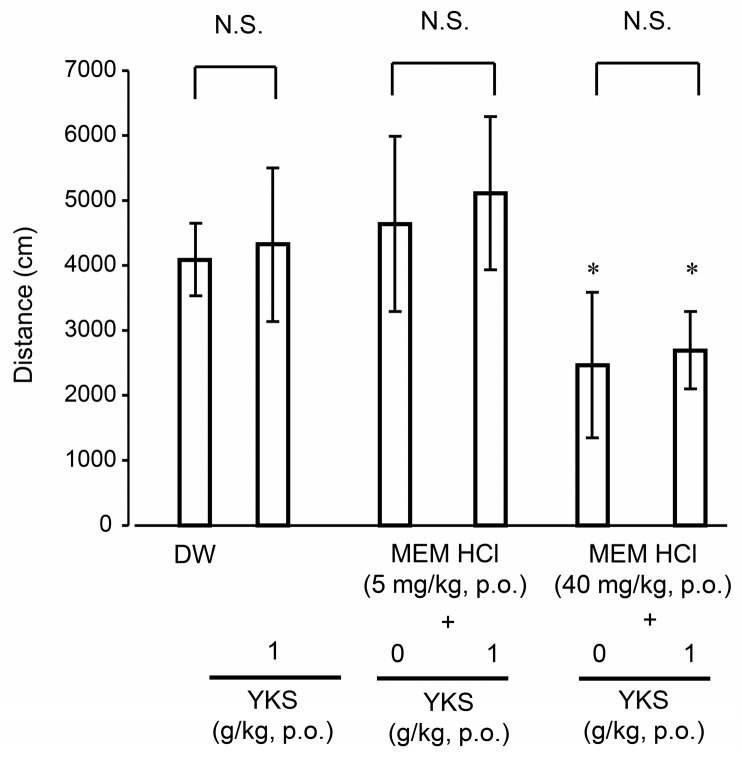
Effects of MEM, YKS, and their combination on locomotor activities in mice in an open-field test. Locomotor activity (total distance traveled in a quadrangular open-field apparatus) for 10 min was measured 1 h after oral administration of MEM hydrochloride (5 or 40 mg/kg), YKS (1 g/kg), or their combination. Data represent mean ± standard deviation (*n* = 10). * *p* < 0.05 versus DW-treated control (Tukey–Kramer test following one-way ANOVA). N.S.: no statistical significance was observed between the two groups.

The effect of MEM, YKS, and their combination on locomotor activities in mice is shown in Figure 5 (one-way ANOVA: *F*_5, 54_ = 10.54, *p* < 0.001). There was no significant difference in activity between the YKS (1 g/kg)-treated group and the control (DW-treated) group. This result is consistent with previous reports showing that YKS did not significantly change behavioral activity in various rodent models of dementia and BPSD [47,48,49,50]. On the other hand, in the MEM-treated groups, while a 5 mg/kg dose did not induce significant changes in motor activity, a 40 mg/kg dose decreased activity significantly (*p* < 0.05). A similar decrease in locomotor activity was reported in mice receiving 30 mg/kg MEM hydrochloride [51]. The locomotor activity in both MEM dose groups was not significantly changed by co-treatment with YKS, suggesting that the combination with YKS does not interfere with the behavioral activity effects of MEM.

## 3. Materials and Methods

### 3.1. Test Drugs

The dry extract of YKS (Lot No. 282059300, Tsumura and Co., Tokyo, Japan) composed of the following seven dried plant materials was used in the present study: Atractylodes lancea rhizome (19.5%; rhizome of *Atractylodes lancea* De Candolle), Poria sclerotium (19.5%; sclerotium of *Wolfiporia cocos* Ryvarden et Gilbertson), Cnidium rhizome (14.6%, rhizome of *Cnidium officinale* Makino), Uncaria hook (14.6%; thorn of *Uncaria rhynchophylla* Miquel), Japanese Angelica root (14.6%; root of *Angelica acutiloba* Kitagawa), Bupleurum root (9.8%; root of *Bupleurum falcatum* Linné), and Glycyrrhiza (7.4%; root and stolon of *Glycyrrhiza uralensis* Fischer). The preparation method of dried extract was in accordance with our previous report [14].

ILQG and GM were supplied from the Botanical Raw Materials Research Department of Tsumura and Co. (Ibaraki, Japan). GA and MEM hydrochloride (Lot No. 041M1252V) were purchased from Sigma-Aldrich (St. Louis, MO, United States).

### 3.2. Animals

Male ddY mice were purchased from Japan SLC (Shizuoka, Japan), and used at age 5–7 weeks for pharmacokinetic and pharmacological (step-through passive avoidance task and open-field test) studies. For the Ca^2+^ influx assay, pregnant female Sprague–Dawley rats were purchased from Charles River Laboratories (Yokohama, Japan), and 18-day-old embryos were used to prepare primary cultured neurons, as described below. All experiments were approved by the Experimental Animal Ethics Committees of Tsumura and Co. (approval number and date: 11-023/June 9, 2011; 11-115/January 23, 2012; 12-015/May 22, 2012; 12-081/October 30, 2012).

### 3.3. Pharmacokinetic Analyses

YKS (1 g) alone, MEM hydrochloride (5 mg) alone, or a combination of the two was dissolved in 10 mL DW, and each preparation was orally administered to 16-h fasted mice at the following doses: YKS, 1 g/10 mL/kg; MEM, 5 mg/10 mL/kg; and YKS + MEM, 1 g YKS and 5 mg MEM/10 mL/kg). Blood was drawn from the abdominal inferior vena cava of mice anesthetized with isoflurane at 0 (pre-administration), 1, 8, and 24 h after oral administration of each drug (*n* = 4,5; time point: the number of animals at each sampling point in each group was designed with *n* = 5. However, one of 5 animals assigned 24 h after YKS administration was excluded, as there was a technical mistake on administration) using a heparinized syringe. Plasma was obtained by centrifugation at 1700× *g* for 15 min at 4 °C. After blood sampling, blood vessels were subsequently perfused with saline, and brains were quickly removed, weighed, and stored at −80 °C prior to analysis. Pooled 24-h urine samples were collected from the mice (*n* = 4,5) used for blood and brain sampling at 24 h after administration. All collected samples, including plasma, brain, and urine, were stored at −80 °C until used for analyses.

All frozen samples were thawed at room temperature prior to analysis. Thawed brain was homogenized using a homogenizer (IKA-T10 model; IKA, Staufen, Germany) after the addition of four volumes (*v*/*w*) of purified water. A 150-μL aliquot of brain homogenate, thawed plasma, or urine sample was mixed with 250 μL of acetonitrile plus 150 μL of vincamine (Tokyo Chemical Ind. Co., Tokyo, Japan) as an internal standard to measure the MEM, GM, and ILQG or niflumic acid (Sigma-Aldrich) as an internal standard to measure GA. Calibration curves were prepared using equal volumes of various concentrations of working solution instead of acetonitrile. Solutions were left to stand for 10 min at 4 °C, then centrifuged at 1700× *g* for 15 min at 4 °C. The supernatant (580 μL) was dried at 40 °C under a stream of nitrogen gas. The dried residue was dissolved in 100 μL of 0.2% formic acid containing methanol (30%, *v*/*v*), then an aliquot of 10 μL was injected into a liquid chromatography–mass spectrometer with tandem mass spectrometer (LC-MS/MS) system for quantification of MEM and the three YKS ingredients. The LC-MS/MS system consisted of an API4000 triple quadrupole mass spectrometer (Sciex, Framingham, MA, USA) equipped with an Agilent 1100 system (Agilent Technologies, Santa Clara, CA, USA). The analytical conditions are summarized in Tables S1 and S2.

### 3.4. Pharmacological Experiments

#### 3.4.1. Measurement of Ca^2+^ Influx in Cultured Neurons

Rat primary cultured cortical neurons were prepared from 18-day-old embryos, and NMDA-induced Ca^2+^ influx in the cultured neurons was measured using Fluo 4-AM as the Ca^2+^ indicator, as previously described [20]. In brief, the cells were cultured for two weeks in 96-well plates, then loaded with 5 μmol/L Fluo 4-AM (Dojindo, Kumamoto, Japan) in a loading buffer at 37 °C for 60 min. Loading was terminated by removing the loading buffer, and then 100 μL of the recording buffer (Dojindo) was added to each well to measure the pre-value. Next, an equal volume of recording buffer containing NMDA (final concentration of 300 μmol/L; Sigma-Aldrich) or NMDA plus each test substance (final concentration of 10–100 μmol/L MEM, 0.1 μmol/L ILQG, or a combination of MEM and ILQG) was added to induce Ca^2^^+^ influx reaction for 3 min. The fluorescence intensities of Fluo-4 before (prevalue) and 3 min after addition of the recording buffer were measured at wavelengths of excitation (485 nm) and emission (518 nm) using the Infinite M200 plate reader (Tecan, Grödig, Austria). Blank intensity data was determined in the same conditions without cultured cells. The specific fluorescence intensity was calculated by subtracting the corresponding blank intensity. The 3-min Ca^2^^+^ influx data was expressed as relative fluorescence intensity against the pre-value.

#### 3.4.2. Step-Through Passive Avoidance Task

Learning and memory functions in mice were evaluated by a step-through passive avoidance task (Neuroscience Inc., Tokyo, Japan) according to the same procedure reported previously [52]. In brief, in the acquisition trial, as soon as the animal placed in the illuminated compartment (130 × 50 × 90 mm, 17 Watts, 1500 lux) entered the dark compartment (170 × 50 × 90 mm), the guillotine door between the two compartments was automatically closed. After the door was closed, the animal automatically received a 1-s foot-shock (0.01 mA, 200 V, 50 Hz) through the floor grids in the dark compartment. The mouse receiving the foot-shock was then immediately returned to a home cage. The retention trial was performed the next day (24 h after the acquisition trial), i.e., the mouse was again placed in the illuminated compartment. The time spent in the illuminated compartment until entering the dark compartment was measured as the latency time. The cut-off time of the latency was 300 s.

Forty-five mice were divided into the following five groups (*n* = 9 per group): control, NMDA, NMDA + MEM, NMDA + YKS, and NMDA + combined MEM + YKS. Distilled water (DW; 10 mL/kg) was orally administered to mice in the control and NMDA groups once a day for 14 days. MEM hydrochloride (5 mg/10 mL/kg), YKS (1 g/10 mL/kg), or MEM + YKS were dissolved in DW, and administered orally to mice in the corresponding groups in the same manner. The acquisition trial was performed 1 h after the final administration on day 14. NMDA (25 mg/10 mL/kg; Sigma-Aldrich) dissolved in saline was intraperitoneally injected to mice in the NMDA, NMDA + MEM, NMDA + YKS, and NMDA + combined MEM + YKS groups 30 min prior to the acquisition trial. Mice in the control group were injected with saline (10 mL/kg) instead of NMDA in the same procedure.

#### 3.4.3. Open-Field Test

An open-field test was used to evaluate the motor activity of mice, as previously reported [47]. In brief, a mouse was placed in the center of a quadrangular open-field apparatus (500 × 500 mm, Neuroscience Inc.), and its behavior was monitored using a charge-coupled device video camera (video tracking system) for 10 min, and the data were saved on a computer. From the saved data, the total distance traveled (cm) in the open-field for 10 min was calculated using LimeLight software (Neuroscience Inc.).

Sixty new naive mice were used for this test. Animals were divided into the following six groups (*n* = 10 per group): control (10 mL/kg DW), MEM hydrochloride (5 mg/kg), MEM hydrochloride (40 mg/kg), YKS (1 g/kg), MEM hydrochloride (5 mg/kg) + YKS (1 g/kg), and MEM hydrochloride (40 mg/kg) + YKS (1 g/kg) groups. Each dose of test substance or mixtures was dissolved in 10 mL of DW. Mice received a single oral administration of each drug or vehicle, and their motor activities were measured for 10 min after 1 h of the administration.

### 3.5. Statistical Analysis

Data from the pharmacokinetic analyses and pharmacological experiments of Ca^2+^ influx and locomotor activity were represented as mean ± standard deviation. The latency times in the learning and memory experiments were represented as median ± interquartile range. The statistical significance was evaluated by Wilcoxon test, Dunnett’s test, Tukey’s test, or Student’s *t*-test using SAS 9.2 software (SAS Institute, Inc., Cary, NC, United States). One-way or two-way ANOVA was used as required, and is indicated in the figure legends. A significance level was accepted at *p* < 0.05.

## 4. Conclusions

In the present study, we examined the DDIs between MEM and YKS from pharmacokinetic and pharmacological perspectives in mice and cultured neurons. There were no statistically significant differences in the pharmacokinetics and pharmacological studies. These results suggest that there is probably no DDI when MEM and YKS are administered in combination, but more detailed studies are needed in the future. Our findings provide important information for the future studies to better clarify the DDI regarding the efficacy and safety for combined use of these drugs in clinical situations.

## Supplementary materials

Supplementary materials are available online, Table S1: Methods of LC-MS/MS: Ion parameters of test compounds, Table S2: LC-MS/MS Methods: HPLC Conditions.
