# Supplementary material for: Basic Study of Drug-Drug Interaction between Memantine and the Traditional Japanese Kampo Medicine Yokukansan
Source: Molecules. 2018 Dec 29;24(1):115. doi: 10.3390/molecules24010115 (PMC6337661; doi:10.3390/molecules24010115)
Supplement: Supplementary file 1 [file molecules-24-00115-s001.pdf]

Table S1. Methods of LC-MS/MS: Ion parameters of test compounds.

**Compound**	**Q1 (*m/z*)**	**Q3 (*m/z*)**	**DP (volts)**	**CE (volts)**	**CXP (volts)**	**Method no.**[†]
Memantine	180.100	107.2	61	35	20	1
Geissoschizine methyl ether	367.173	144.2	96	45	28	1
Isoliquiritigenin	254.932	118.9	-75	-36	-19	1
Glycyrrhetinic acid	469.273	425.3	-130	-54	-23	2
Vincamine (IS)	355.215	294.3	81	39	26	1
Niflumic acid (IS)	280.826	236.8	-55	-24	-11	2

[†]: Method no. is linked to number described in Table S2. DP; declustering potential, CE; collision energy, CXP; collision cell exit potential.

Table S2. LC-MS/MS Methods: HPLC Conditions.

**Method no.**	**HPLC condition**
1	Column: Ascentis Express RP-amide column (100 × 2.1 mm I.D., 2.7-µm particle size; Supelco, Bellefonte, PA)<br>Mobile phase (A) 0.2 vol % formic acid, (B) acetonitrile<br>Gradient elution program (% B in A):<br>0–12 min, 13–15%; 14 min, 20%; 24 min, 25%; 24.1 min, 65%; 30–33 min, 100%; 33.1–38 min, 13%<br>Other conditions were: flow rate, 0.3 mL/min; column temperature, 40°C
2	Column: YMC-Pack ODS-AQ column (50 × 2.0 mm I.D., 3-µm particle size; YMC Co., Kyoto, Japan)<br>Mobile phase (A) 10mmol/L ammonium acetate, (B) acetonitrile<br>Gradient elution program (% B in A):<br>0–5 min, 55–100%; 7 min, 100%; 7.01–10 min, 55%<br>Other conditions were: flow rate, 0.2 mL/min; column temperature, 40°C
